# Sec61 blockade therapy overrides resistance to proteasome inhibitors and immunomodulatory drugs in multiple myeloma

**DOI:** 10.3389/fonc.2023.1110916

**Published:** 2023-01-27

**Authors:** Antoine Domenger, Daniela Ricci, Véronique Mayau, Laleh Majlessi, Christophe Marcireau, Gilles Dadaglio, Caroline Demangel

**Affiliations:** ^1^ Immunobiology and Therapy Unit, Institut Pasteur, Université Paris Cité, Institut National de la Santé et de la Recherche Médicale (INSERM) U1224, Paris, France; ^2^ Pasteur-TheraVectys Joint Lab, Institut Pasteur, Université Paris Cité, Paris, France; ^3^ Molecular Oncology, Sanofi, Vitry-sur-Seine, France

**Keywords:** multiple myeloma, sec61 translocon, proteasome inhibitors, immunomodulatory drugs, drug resistance, UPR – unfolded protein response

## Abstract

Multiple Myeloma (MM) is an incurable neoplasm of mature B cells and the second most prevalent hematological malignancy worldwide. While combinations of proteasome inhibitors like bortezomib (Bz) and immunomodulators (IMiDs) like lenalinomide (Len) are generally effective in newly diagnosed patients, some do not respond to this first-line therapy, and all others will eventually become drug resistant. We previously reported that inhibiting the Sec61 translocon with mycolactone synergizes with Bz to induce terminal unfolded protein response in MM cells, irrespective of their resistance to proteasome inhibition. Here, we examined how Sec61 blockade interferes with IMiD action and whether it overrides resistance to Len. With this aim, we knocked out the IMiD target CRBN in the MM1S cell line and a Bz-resistant subclone to generate Len- and Len/Bz-resistant daughters, respectively. Both the Len- and Len/Bz-resistant clones were susceptible to mycolactone toxicity, especially the doubly resistant one. Notably, the synergy between mycolactone and Bz was maintained in these two clones, and mycolactone also synergized with Len in the two Len-susceptible ones. Further, mycolactone enhanced the therapeutic efficacy of the Bz/Len combination in both mice engrafted with parental or double drug resistant MM1S. Together, these data consolidate the interest of Sec61 blockers as new anti-MM agents and reveal their potential for treatment of refractory or relapsed MM.

## Introduction

Multiple myeloma (MM) is a hematological malignancy of plasma cells, the mature B lymphocytes producing immunoglobulins. Proteasome inhibitors (PIs) and immunomodulatory drugs (IMiDs) such as Bz and Len are the backbone agents of a MM combination therapy significantly prolonging patient survival (1). However, newly diagnosed patients can display primary refractory MM and most of those initially treated will eventually develop drug-resistant MM, with a poor prognosis (2, 3).

The anti-MM activity of Bz primarily relies on its ability to induce a terminal unfolded protein response (UPR) *via* toxic accumulation of misfolded immunoglobulins in the endoplasmic reticulum (ER) (4). For its part, Len targets the Cereblon (CRBN) component of an E3 ubiquitination complex, provoking the selective degradation of the tumor pro-survival transcription factors IKZF1s, and leading to MM cell cycle arrest and apoptosis (5). While the molecular basis of MM resistance to PIs is complex, that of IMiD is typically associated with altered CRBN expression (6).

Using mycolactone (Myco) as a model inhibitor, we recently reported that Sec61 - the channel mediating secretory protein import into the ER - is a therapeutic vulnerability in MM (7). By preventing the translocation of newly synthesized secreted and transmembrane proteins into the ER, Myco provokes their cytosolic degradation by the proteasome (8–11). In MM cell lines and patient-derived tumors, Sec61 blockade by Myco triggered an atypical, pro-apoptotic ER stress response synergizing with Bz for induction of MM cell death *in vitro* and *in vivo* (7). Using a Bz-resistant version of the model MM cell line MM1S (12), we showed that Sec61 blockade overrides MM resistance to PIs. Here, we investigated the therapeutic interest of combining Sec61 inhibition to Len and Len+Bz combinations, in both chemo-naïve and -resistant MM.

## Method

### Reagents

Myco was purified from *M. ulcerans* bacterial pellets (strain 1615) then quantified by spectrophotometry and stored in ethanol at -20°C protected from light (13). For *in vivo* experiments, a 4 mM stock was diluted in a NaCl solution (0.9% w/v) immediately before injection in animals. For *in vitro* experiments, a 1000 x working solution was prepared by dilution of the ethanol stock in DMSO and stored at -20°C, then thawed and diluted in culture medium immediately before use (14). Bz purchased from Alfa Aesar (#J60378) was resuspended in DMSO to yield a 10 mM solution stored at -20°C. Len purchased from Sigma-Aldrich (#SML2283) was resuspended in DMSO to yield a 40 mM stock solution stored at -20°C. Bz and Len stock solutions were thawed and diluted in culture medium immediately before use. Thapsigargin (Tocris, #1138) was resuspended in DMSO to yield a 10 mM solution stored at -20°C.

### Lentiviral vector production

Our plasmid vector was produced by cloning the CRBN specific single guide RNA (CCTTTGCTGTTCTTGCATAC) (5) into the backbone plasmid LentiCRISPRv2GFP (Adgene#82416) following the provided protocol. The Non-integrative lentiviral vector (LV) was produced in Human Embryonic Kidney (HEK) 293-T cells, as previously detailed (15). LV particles were generated by transient calcium phosphate tripartite co-transfection of HEK 293-T cells with: (i) the vector plasmid, (ii) a Vesicular Stomatitis Virus (VSV)-G Indiana envelope plasmid, and (iii) a lentiviral encapsidation pD64V plasmid. Supernatants were harvested at 48h, clarified by centrifugation at 2500 rpm at 4°C for 10 min and stored at -80°C. The LV titer was determined as Transduction Units (TU)/ml by transducing HEK 293-T cells, as previously detailed (16).

### MM1S cell lines

The MM1S cell line used in this study was a gift from J.-C.B. (Fayon et al, 2020) authenticated by Eurofins Genomics. In pilot experiments, we found that the Len treatment conditions inducing maximal mortality and proliferation arrest in this cell line were 80 µM Len for 120h. A daughter line of MM1S with stable and >15x increased resistance to Bz was generated previously, by serial subculture of MM1S in the presence of increasing doses of Bz (7). We generated Len-resistant versions of each cell line by CRISPR-Cas9 knock out of the *CRBN* gene. Cells were transduced with LV particles produced as described above. Selection of *CRBN* ko cells was performed by addition of 80 µM Len to the cell culture medium. All cell lines (parental MM1S, single and double resistant daughters) were tested mycoplasma negative. They were cultured in RPMI 1640 medium supplemented with 10% fetal bovine serum (FBS, Dominique Dutcher #S1810-500), 100 Units/mL penicillin + 100 µg/mL streptomycin (Gibco, #15140-122).

### Viability assay

Exponentially growing cells were plated in 96 well plates at a density of 3.10^4^ cells/well, then treated as indicated and incubated at 37°C. Cell viability was assessed by Annexin V exposure and PI incorporation using the FITC-Annexin V/PI kit from Miltenyi Biotec (#130-092-052) following the manufacturer’s protocol. Live cells were characterized as Annexin V^-^ PI^-^, apoptotic cells as Annexin V^+^ PI^−^ and dead cells as Annexin V^+^ PI^+^ and Annexin V^−^ PI^+^.

### Western blot analyses

Following drug treatment, 2.10^6^ cells were harvested and solubilized at 10^8^ cells/ml for 15 min in ice-cold lysis buffer containing 1% Nonidet P-40, 1% n-dodecyl-{beta}-D-maltoside, 20 mM Tris-HCl, pH 7.5, 150 mM NaCl, 1 mM MgCl2, and 1 mM EGTA in the presence of inhibitors of proteases and phosphatases (10 µg/ml leupeptin, 10 µg/ml aprotinin, 1 mM Pefabloc-sc, 50 mM NaF, 10 mM Na4P2O7, and 1 mM NaVO4). For immunoblot analyses, loadings were normalized based by total amount of proteins. Proteins were then separated by sodium dodecyl sulfate polyacrylamide gel electrophoresis (SDS-PAGE) using NuPAGE Bis-Tris gels (Thermo Fisher Scientic) and transferred on nitrocellulose membranes (iBlot 2^®^ gel transfer Stacks Nitrocellulose system from Invitrogen). Immune blotting was carried out by overnight incubation at 4°C with the primary antibodies anti-CRBN (SIGMA HPA045910) and anti-IKZF1 (Cell Signaling #14859). After washing, the membranes were incubated with HRP conjugated anti-rabbit IgG secondary antibodies (Santa Cruz, sc-2004, Biolegend, 405405) for 45 min at room temperature. Detection of proteins was performed with the enhanced chemical luminescence (ECL) method using the ECL Prime Western Blotting Reagent and image were acquired on an ImageQuant LAS 4000 Mini (GE Healthcare).

### Gene expression analyses

Following drug trteatment, 2.10^6^ cells were harvested and lysed in Trizol (Qiagen). Chloroform was added to the trizol lysates, and the mix was then centrifuged for 15 min at 15,000 rcf and 4°C. After centrifugation, the aqueous phase was recovered and mixed with 1.5 volume of ethanol. Total mRNAs were then extracted using RNeasy Plus Mini Kit (Qiagen) according to the manufacturer’s procedure and reverse-transcribed into cDNAs using High Capacity cDNA Reverse Transcription Kit (BD Bioscience) from 1 µg total mRNA according to the manufacturer’s recommendations. The levels of transcription of the mRNAs coding for the genes of interest were assessed using SyberGreen (Power SYBR Green PCR Master Mix, applied biosystem ref: 4367659) and the following primers synthesized by Eurofins genomics: ATF4 (forward), CACCGCAACATGACCGAAAT; ATF4 (reverse), GACTGACCAACCCATCCACA; CHOP (forward), GCACCTCCCAGAGCCCTCACTCTCC; CHOP (reverse), GTCTACTCCAAGCCTTCCCCCTGCG; sXBP1 (forward), GGTCTGCTGAGTCCGCAGCAGG; sXBP1 (reverse), GGGCTTGGTATATATGTGG; BiP (forward), CGAGGAGGAGGACAAGAAGG; BiP (reverse), CACCTTGAACGGCAAGAACT. Quantitative PCR conditions used were: 50°C 2 min, 95°C 10 min, 95°C 15 s (40 cycles) and 60°C for 1 minute. The relative quantification was calculated by the 2^-ΔΔCT^ method and the 18S mRNA was used as endogenous control.

### Proliferation assay

Cell proliferation was assessed using BrdU incorporation. Briefly, 2.10^5^ cells were treated as indicated, then exposed to BrdU for 4h. The proportion of BrdU^+^ cells was assessed using the FITC BrdU flow kit (BD Pharmingen 559619) according to the supplier’s instructions.

### Mouse experiments

NOD.Cg-*Prkdc^scid^ Il2rg^tm1Wjl^*/SzJ (NSG, stock number: 005557) mice were purchased from the Jackson laboratory and were used between 6 to 12 weeks of age. Mice were housed at animal facilities of the Institut Pasteur under specific pathogen–free conditions with food and water ad libitum. 3.10^6^ MM1S cells (parental or doubly drug resistant) were subcutaneously injected in the right flank of the animals in 200 µL of PBS. After 24 h, mice were randomly assigned to 4 groups and 6 days later, each group was randomly assigned a treatment. Drugs were administered every 3.5 days: Myco (0.3 mg.Kg^-1^) and Bz (0.3 mg.Kg^-1^) *via* the intraperitoneal route and Len (50 mg.Kg^-1^) by oral gavage. Tumor growth was assessed daily by unblinded measurement of tumor size with a digital caliper. Data are presented as the average of two perpendicular diameters (millimeters). Mice were sacrificed when the tumor diameter reached 20 mm or whenever the animal shows clinical sign of pain according to ethical guidelines. At the end of *in vivo* experiments, mouse blood was sampled. Serum was isolated by centrifugation of coagulated blood at 200 g for 10 min at 4°C, and serum levels of alanine aminotransferase (ALT) were assessed with the Mouse ALT ELISA Kit (Abcam ab282882) according to the supplier’s instructions.

### Synergy scores

Synergy between drugs was assessed with the *combenefit* software (17) which calculates scores based on the Loewe additivity model using the dose response of each drug. Loewe synergy score are defined as S_LOEWE_ = Y_obs_ -Y_Loewe_, where Y_obs_ is the observed effect of the combination and Y_Loewe_ is the theoretical effect of the combination. Therefore, a S_LOEWE_ > 0, shows that drugs act in synergy. On the contrary, S_LOEWE_ <0, depicts an antagonist effect of the drugs. S_LOEWE_ were plotted as heatmaps and statistical significance analyzed by one-sample Student’s t-test.

### Statistical analyses

Other statistical treatments and graphical representations were performed with the Prism software (v8.4.3, GraphPad, La Jolla, CA) and values of P ≤ 0.05 were considered significant. Detailed information on the statistical test used and number of replicates is provided in figure legends.

## Results

**Figure 1:**
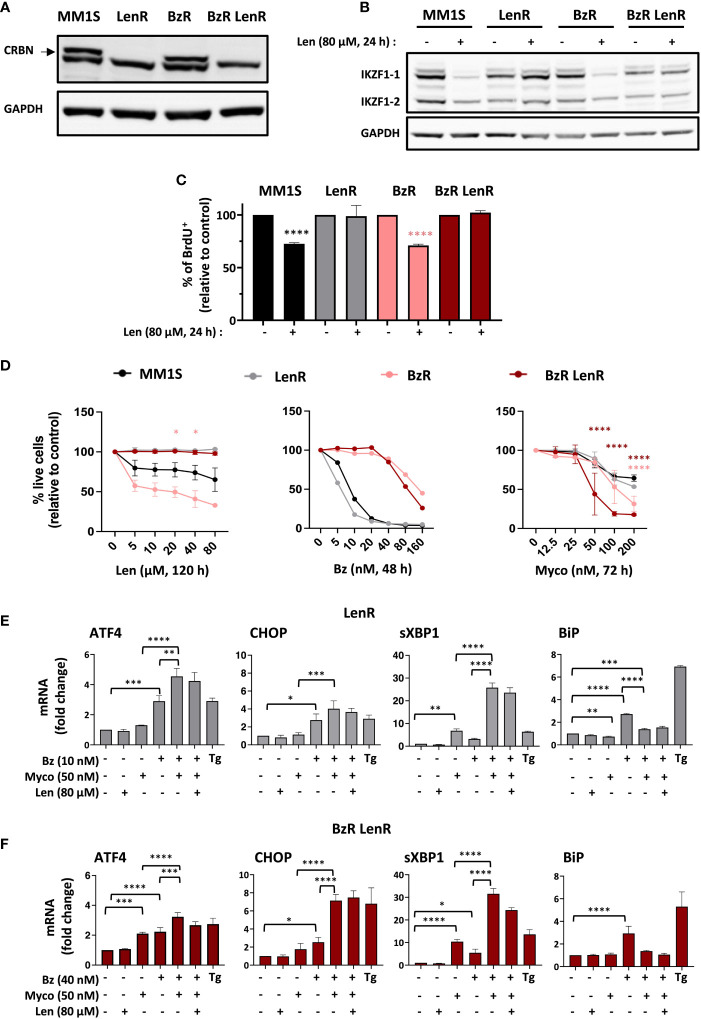
Sec61 blockade overrides MM resistance to Len. **(A)** Expression of CRBN by MM1S (parental) and BzR, LenR, BzR LenR daughters. **(B)** Expression of IKZF1.1/2 in the 4 cell lines treated with 80 µM Len or vehicle for 24h. In **(A, B)**, protein levels were assessed in cell lysates and quantified relatively to GADPH levels. **(C)** Proportion of proliferating cells, as assessed by BrdU incorporation, in the 4 cell lines treated as in **(B)**. Data are Mean % ± SD of technical triplicates relative to vehicle controls, from one experiment representative of two. **(D)** Comparative analysis of drug susceptibility of the four cell lines. Each cell line was treated with Len (120h), Bz (48h) or Myco (72h), and cell viability was assessed by exposure of Annexin V and incorporation of PI. Data are Mean % of live cells (relative to controls) from the cumulated results of 3 (Len), or 2 independent experiments (Bz and Myco). Differences between daughter cell lines and parental MM1S by one-way Anova followed by Tukey’s multiple comparison test (*p<0.05, ****p<0.0001). **(E)** In LenR MM1S cells treated as indicated with the different drugs or vehicle for 6h, mRNA levels of ATFA, CHOP, sXBP1 and BiP were quantified by qPCR. Thapsigargin (Tg, 2 µM, 6 h) was used as positive control. Shown mRNA data are Mean fold changes (2^-ΔΔCT^) ± SD, relative to untreated controls from the cumulated data of 3 independent experiments. **(F)** Same as **(E)** with BzR LenR MM1S cells. In **(C)**, **(E, F)**, differences between treated cells and controls by two-way Anova followed by Tukey’s multiple comparison test (*p < 0.05; **p < 0.01; ***p < 0.001, ****p < 0.0001).

Len-resistant (LenR) and double, Len- and Bz-resistant (BzR LenR) cell lines were generated by CRISPR/Cas9 knockout of *CRBN* in MM1S and BzR daughter (7), respectively. Both were fully defective for production of CRBN (**Figure 1A**) and protected against Len-induced degradation of IKZF1.1 and IKZF1.2 (**Figure 1B**). In both the parental MM1S and BzR cell lines, Len had a marked anti-proliferative effect after 24h of treatment (**Figure 1C**). On the opposite, the growth of LenR and BzR LenR MM1S was unaffected by Len (**Figure 1C**). While 100% mortality was achieved in MM1S cells exposed to 80 nM Bz for 48h, the cytotoxicity of Myco only manifested after 72h at concentrations ≥ 50 nM (**Figure 1D** and 7). The anti-MM activity of Len was even slower, in accordance with previous reports (5). A maximal viability loss of 50% was obtained after a 120h treatment with 80 µM Len in parental and BzR MM1S cells, from which CRBN ko cells were protected (**Figure 1D**, left). In contrast, MM1S and its LenR daughter were equally sensitive to Bz and Myco (**Figure 1D**, middle and right). It is interesting to note that BzR LenR and BzR MM1S cells displayed an increased sensitivity to Myco at the highest tested concentrations, compared to parental MM1S cells (**Figure 1D**, right). Blocking Sec61 thus overcomes MM cell resistance to IMiDs, and even more if it is combined with resistance to PIs.

The clinical efficacy of PIs primarily relies on their capacity to trigger transition from adaptive to terminal UPR in MM, through induction of the Activating Transcription Factor 4 (ATF4) and its pro-apoptotic target C/EBP homology protein (CHOP). Other hallmarks of Bz activity include elevated splicing of X-box binding protein 1 (XBP1) mRNA into transcriptionally active sXBP1 and induction of the ER-resident chaperone BiP, a master regulator of the UPR controlling the threshold of apoptosis induction (18). We previously reported that Myco synergizes with Bz by inducing an atypical UPR marked by hyper-activation of ATF4/CHOP signaling and XBP1 splicing, in the absence of BiP upregulation (7). **Figure 1E** shows that the distinctive ER stress signatures of Bz, Myco and Bz+Myco were maintained in LenR MM1S cells (**Figure 1E**). In comparison, BzR LenR MM1S displayed a relatively more important induction of *ATF4* gene expression in response to Myco treatment (**Figure 1F**). In none of the two CRBN ko cell lines did a co-treatment with Len interfere with these stress marks. Therefore, both the proteotoxic effects of Sec61 blockade and synergy with proteasome inhibition withstand MM resistance to Bz and Len.

**Figure 2:**
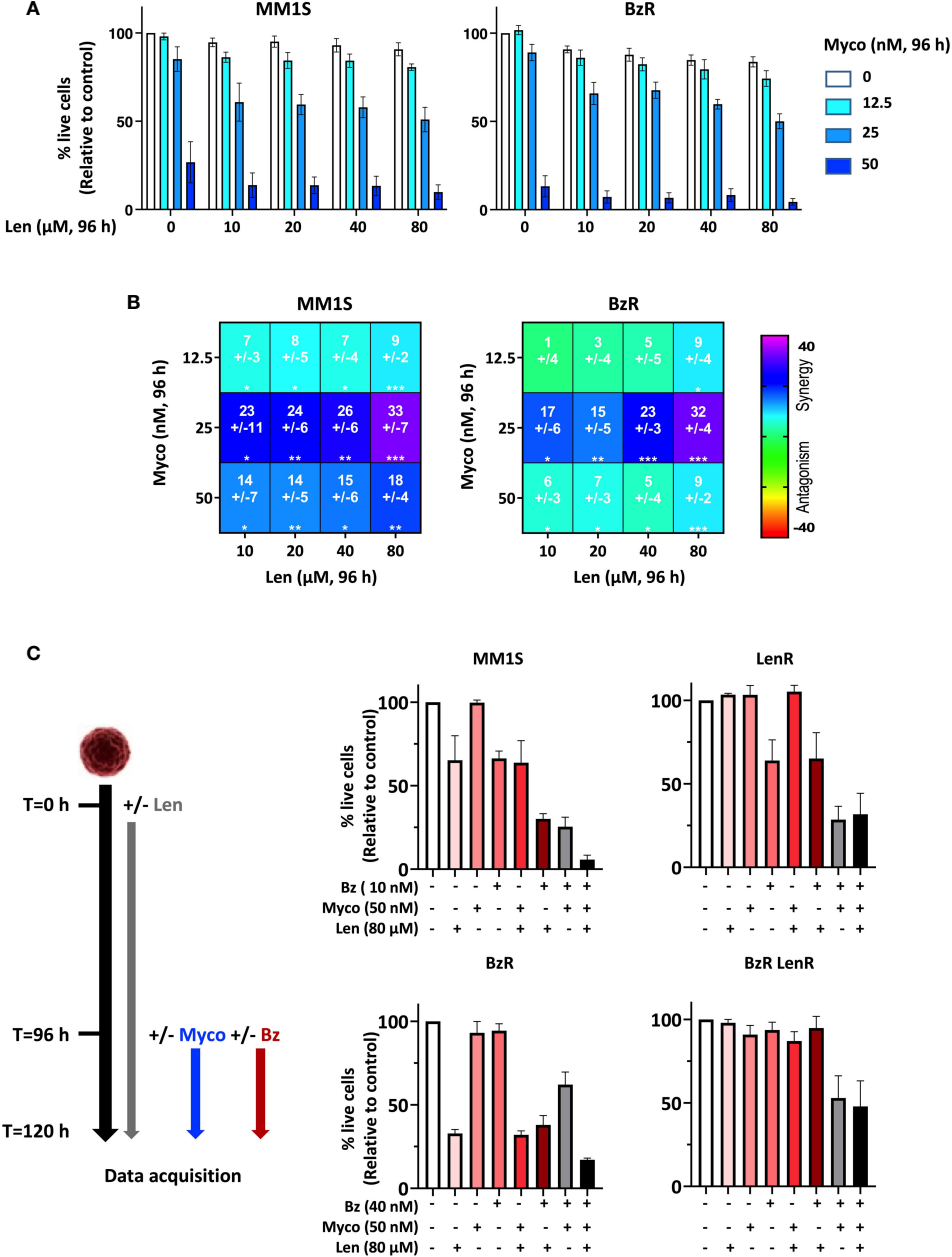
Anti-MM efficacy of drug combinations including a Sec61 blocking agent. **(A)** Effect of Len+Myco combinations on the viability of MM1S (parental) and BzR daughter. Each cell line was treated as indicated with the different drugs or vehicle for 96h, and cell viability was assessed by exposure of Annexin V and incorporation of PI. Data are Mean % of live cells (relative to controls) from the cumulated results of 2 independent experiments with 3 technical replicates. **(B)** Synergy between Len and Myco in parental MM1S (left) or its BzR daughter (right), when treated as in **(A)**. The Loewe synergy score was calculated from the Data showed in **(A)**. Statistical significance was established by Student’s T test (*p < 0.05; **p < 0.01; ***p < 0.001, ****p < 0.0001). **(C)** *Left:* Diagram illustrating the experiment conditions. *Right:* Differential susceptibility of the four cell lines to mono-, bi- and tri-therapies (Right). Cells were treated as indicated with the different drugs or vehicle and cell viability was assessed by exposure of Annexin V and incorporation of PI. Data are Mean % of live cells (relative to controls) from the cumulated results of 3 independent experiments.

To determine if Myco interferes with the anti-MM activity of Len, we next assessed the cytotoxicity of Len/Myco combinations in MM1S and its BzR version (**Figure 2A**). After 96h of exposure, Len marginally affected MM cell viability while Myco displayed a clear dose-dependent cytotoxicity. Notably, in both cell types the two drugs synergized to induce MM cell death (**Figure 2B**), highlighting the interest of combining Sec61 blockers with IMiDs in MM treatments.

We went on to assess the impact of bi- and tri-therapies in both parental MM1S and its single- and double-drug resistant daughters. Because Len takes 120h to display cytotoxicity, cells were treated with Len alone for 96h, then Myco and Bz were added for the next 24h. In all cell lines, the Myco/Bz combination was highly cytotoxic (**Figure 2C**), confirming our previous findings obtained with parental and BzR MM1S (7), and extending them to their LenR and BzR LenR counterparts. Notably, adding Len to the Myco/Bz combination further increased the induction of apoptosis in the two Len-susceptible MM1S cell lines. Altogether, these results consolidated the potential of blocking Sec61 in drug-resistant MM.

**Figure 3:**
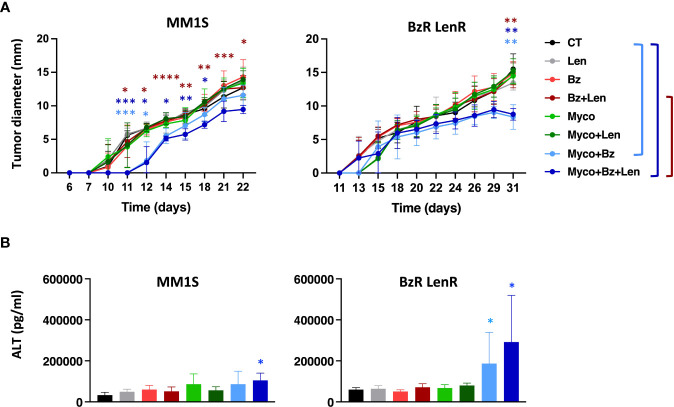
*In vivo* evaluation of Sec61 blockade in treatment of drug-sensitive and -resistant MM. **(A)** NSG mice (N = 6) were injected subcutaneously with 3.10^6^ MM1S (parental) or its BzR LenR daughter at day 0. Seven days later, mice were treated with DMSO, Bz (0.5 mg.Kg^-1^), Myco (0.3 mg.Kg^-1^) and/or Len (50 mg.Kg^-1^) every 3.5 days. Myco and Bz were administrated by the intra-peritoneal route and Len by oral gavage. Tumor growth was followed by daily measurement of the tumor diameter. Data are Mean tumor diameters ± SD and represent cumulative data from 2 independent experiments with 3 mice/group. Difference between groups were analyzed with Tukey’s multiple comparison test using Two-way Anova, with mixed effect model for each time points: *p<0.05; **p<0.01; ***p<0.001. Only significant differences are shown. **(B)** Sera of the mice used in **(A)** were sampled at the end of the experiment and ALT levels assessed by ELISA. Differences between treated groups and control by one-way Anova followed by Tukey’s multiple comparison test (*p < 0.05; **p < 0.01; ***p < 0.001, ****p < 0.0001).

To evaluate the interest of adding Myco to the Bz/Len bi-therapy *in vivo*, we engrafted MM1S on the one hand, and its BzR LenR counterpart on the other hand, in immunodeficient NOD/SCID/IL2rγnull (NSG) mice as described in Domenger et al. (7). Mice were randomized in eight groups receiving Len, Myco and Bz as single, double, or triple drug combinations twice weekly. In the conditions used, none of the monotherapies did influence the growth of MM1S cells (**Figure 3A**). In contrast, the Bz/Myco combination significantly delayed the progression of both MM1S and its BzR LenR version. In both systems, the tri-therapy was significantly superior to the clinically used Bz/Len combo. The Myco/Bz/Len treatment, and Myco/Bz to a lower extent, nevertheless induced toxic effects, with elevated levels of serum ALT suggesting hepatotoxicity (**Figure 3B**).

## Discussion

Using the Sec61 inhibitor Myco and a combination of MM cell lines, patient-derived tumors and xenograft mouse models of disease, we previously demonstrated that pharmacological blockade of Sec61 triggers MM cell-selective apoptosis through induction of ER stress (7). Inhibiting Sec61 also impaired MM cell secretion of immunoglobulins and surface expression of pro-survival receptors. Moreover, it synergized with Bz for MM cell killing and this synergy extended to B cell acute lymphoblastic leukemia. Collectively, these data established the translational potential of Sec61 blockers as novel anti-MM agents. Beyond MM, they uncovered the interest of targeting both the translocon and the proteasome in proteostasis-addicted tumors (7). This study also revealed that Sec61 blockade kills MM cells irrespective of their innate or acquired resistance to Bz (7). In addition to MM1S, Myco treatment induced apoptosis in the JIM3 and KMS11 cell lines, which display a relatively higher resistance to Bz. Myco also showed potent cytotoxicity in MM cells isolated from 6 patients, with variable resistance to Bz. By generating a Bz-resistant daughter of MM1S, we could formally demonstrate that the anti-MM activity of Myco overrides resistance to proteasome inhibition.

Since the current standard of MM care combines PIs with IMiDs, it was important to determine how Sec61 blockade interferes with- and whether it overrides resistance to- IMiDs. We show in the present study that both the toxicity of Myco and synergy with Bz operate in LenR and BzR LenR MM1S cells. Interestingly, at concentrations ≥ 200 nM Myco was more effective in BzR cells, and even more in BzR LenR cells, than in the parental cell line. In addition to activating the UPR, we reported previously that Myco triggers pro-apoptotic oxidative stress responses through depletion of intracellular glutathione pools (19). Interestingly, recent proteomic studies of MM cells have associated acquired resistance to PIs with the generation of important oxidative stress (20, 21). This may render BzR cells more susceptible to the lethal oxidative activity of Myco. Furthermore, deletion of CRBN was shown to promote PERK signaling (22), providing a possible explanation for the superior susceptibility of BzR LenR cells, compared to BzR cells, to Myco-mediated induction of terminal UPR.

Notably, we also observed a synergy between Myco and Len in parental and BzR MM1S. Furthermore, Myco enhanced the efficacy of the PI/IMiD bitherapy in mice engrafted with MM1S cells, irrespective of their resistance to PIs and IMIDs. While confirmatory studies with more MM cell lines will be needed, these data support the interest of further evaluating Sec61 blockers in MM drug combinations. They strongly suggest that MM patients developing resistance to PIs and/or IMiDs will respond to Sec61 blockade therapy.

While a useful tool to study the consequences of Sec61 blockade *in vitro* and *in vivo*, Myco is a complex natural product whose structure and physicochemical properties are incompatible with drug development (23, 24). Furthermore, its broad-spectrum inhibitory activity on Sec61 substrates confers Myco with toxic effects on non-cancerous cells that limit its therapeutic window (8, 10, 25). Importantly, newly generated small molecule inhibitors of Sec61 are currently being evaluated in Oncology phase 1 trials, paving the way to the first generation of drugs targeting protein translocation (26). The work presented here suggests that blocking Sec61 is a promising therapeutic avenue for the treatment of refractory or relapsed MM.

## Data availability statement

The original contributions presented in the study are included in the article/supplementary material. Further inquiries can be directed to the corresponding authors.

## Ethics statement

The animal study was reviewed and approved by the CETEA ethics committee number 0068 (Institut Pasteur, Paris, France, DAP210001) and received the approval of the French Ministry of Higher Education and Research.

## Author contributions

AD, DR and VM performed the experiments. LM generated the lentiviral particles for CRISPR/Cas9 knock out of CRBN. AD, CM, GD, and CD designed the experiments. Data interpretation and writing of the manuscript were performed by AD and CD. All authors contributed to the article and approved the submitted version.
